# Molecular Surveillance of ESBL and Carbapenemase Genes in Gram-Negative Bacterial Pathogens Isolated from Various Clinical Samples Collected from Northern Region of United Arab Emirates

**DOI:** 10.3390/microorganisms13081880

**Published:** 2025-08-12

**Authors:** Premalatha Ragupathi, Vaneezeh Khamisani, Aisha Fadila Sadiq, Mariam Aliyu Mobiddo, Nasir Parwaiz, Sovan Bagchi, Nazeerullah Rahamathullah

**Affiliations:** 1 Department of Biomedical Sciences, College of Medicine, Gulf Medical University, Ajman 4184, United Arab Emirates; premalatha.r@gmu.ac.ae (P.R.); vaneezehkhamisani@gmail.com (V.K.); 2018bm01@mygmu.ac.ae (M.A.M.); dr.sovan@gmu.ac.ae (S.B.); 2 Thumbay Laboratory, Thumbay University Hospital, Gulf Medical University, Ajman 4184, United Arab Emirates; dr.parwaiz@thumbayhospital.ae; 3 Thumbay Research Institute for Precision Medicine, College of Medicine, Gulf Medical University, Ajman 4184, United Arab Emirates

**Keywords:** Gram-negative bacteria, multidrug resistant genes, extended spectrum of beta lactamase, *bla_TEM_*, *bla_CTX-M_*, *bla_SHV_*, *bla_NDM_*, *bla_OXA-48_*, *bla_IMP_*

## Abstract

The aim of this study was to explore the prevalence of ESBL and carbapenemase genes in Gram-negative bacteria isolated from various clinical samples collected from northern regions of UAE. In total 3670 clinical samples were obtained from patients attending various hospitals and clinics in the northern regions of the UAE. All the samples underwent routine bacterial culture examination, and their antibiotic sensitivity patterns mainly on beta-lactam and carbapenem resistance in Gram-negative bacteria. Molecular detection of ESBL and carbapenemase genes (*bla_CTX-M_*, *bla_TEM_*, *bla_SHV_*, *bla_NDM_*, *bla_IMP_*, and *bla_OXA-48_*) was performed on them. A total of 249 MDR Gram-negative bacteria (*E. coli*, *K. pneumoniae*, *P. aeruginosa*, *P. mirabilis* and *A. baumannii*) were isolated. The genes *bla_CTX-M_*, *bla_TEM_*, and *bla_SHV_* were detected in all the MDR isolates. Among them, the *bla_CTX-M_* was predominant especially in *E. coli*. The *bla_NDM_* and *bla_IMP_* were detected in a few *K. pneumoniae* and *A. baumannii*. The genes combination *bla_CTX-M+TEM_* and *bla_CTX-M+SHV_*, *bla_CTX-M+SHV_*, *bla_TEM+SHV_*, and *bla_TEM+NDM_* were detected mostly in *K. pneumoniae* and *E. coli*, and few *A. baumannii*. The gene combination *bla_CTX-M+TEM+SHV_* and *bla_CTX-M+TEM+SHV+IMP_* were also detected in few *E. coli*, *P. aeruginosa*, and *A. baumannii*. The current findings highlight the importance of molecular detection of ESBL and carbapenemase genes to emphasize monitoring and controlling the development of MDR bacterial pathogens.

## 1. Introduction

Antimicrobial resistance (AMR) is a serious public health challenge worldwide and poses a significant threat to the resilience of the global healthcare system, animal production, and environmental health. It increases the mortality and morbidity rates and inflates the healthcare expenditure [1]. Globally, over 7 million people die every year due to multidrug-resistant (MDR) bacterial infections. It is estimated that 10 million people will die from antimicrobial-resistant infections by 2050 [2]. Overprescribing, improper use of broad-spectrum antibiotics, and not adhering to prescriptions are some of the leading causes of emergence of AMR bacteria [3,4]. Gram-negative bacteria are more prone to developing antibiotic resistance than Gram-positive bacteria and due to the enhanced use of beta-lactam antibiotics, bacterial pathogens have developed the ability to produce the enzyme beta-lactamase using plasmids which counteract the beta-lactam compound [5,6].

Rapid spread of antibiotic resistance genes among the Gram-negative bacteria facilitated by mobile genetic elements, such as plasmids and transposons, is responsible for resistance to a broad spectrum of antibiotics and plays a pivotal role in the dissemination of AMR. The spread of plasmid mediated beta-lactamase in Gram-negative bacterial pathogens is known as extended-spectrum beta-lactamase (ESBL). Molecular classification of the β-lactamases divides them into A, B, C, and D enzymes, with the genes *bla_TEM_*, *bla_SHV_*, and *bla_CTX-M_* grouped under class A [7]. Over 172 CTX-M, 223 TEM, and 193 SHV types have been identified and listed in public databases [8].

ESBL producers have emerged as a major source of resistance among Gram-negative bacteria, predominantly in *Escherichia coli* and *Klebsiella pneumoniae*, thereby becoming a global concern [8,9]. *K. pneumoniae* strains that produce ESBLs and carbapenemase have a worldwide distribution and cause multidrug-resistant (MDR) infections in many countries [10]. Carbapenems are one of the few drugs that are useful for the treatment of MDR Gram-negative bacterial infections. The emergence of carbapenem resistance in the family *Enterobacteriaceae* leads to serious public health concern due to the large spectrum of resistant genes and lack of therapeutic options [11,12]. Currently carbapenem resistance non-fermenting bacteria such as *Acinetobacter baumannii* and *Pseudomonas aeruginosa* and other Gram-negative bacteria have received more attention. A number of reports from worldwide sources have stated that carbapenem-resistant genes, such as classes A (e.g., KPC, GES, IMI, SME), B (e.g., IMP, VIM, NDM, GIM), and D (e.g., OXA-23, OXA- 48), are frequently detected in *Enterobacteriaceae* and non-fermentative bacteria, mainly *A. baumannii*, *P. aeruginosa*, and others [13].

Furthermore, ESBL-developing species show co-resistance to many other groups of antibiotics, limiting therapeutic options [14,15,16]. The rising prevalence of Gram-negative bacterial infections in the UAE constitutes a major public health threat, amplified by the increasing burden of MDR organisms in both community and healthcare environments. A national surveillance program, spanning 2010 to 2021 and involving over 660,000 clinical isolates across the country, identified *E. coli* (27.8%), *K. pneumoniae* (11.4%), and *Pseudomonas aeruginosa* (5.9%) as leading pathogens among priority AMR agents [17,18]. However, there is still a scarcity of data on the prevalence of ESBL and carbapenemase-producing genes in Gram-negative fermentative bacteria such as *E. coli* and *K. pneumoniae*, and non-fermentative bacteria including *P. aeruginosa*, *Acinetobacter baumannii*, and *Proteus mirabilis* isolated from various clinical samples in the UAE. Although some data have been published from this region and neighboring countries, a limited number of studies are available on the genes *bla_TEM_*, *bla_SHV_*, *bla_CTX-M_*, *bla_NDM_*, *bla_IMP_*, and *bla_OXA-48_*. However, no detailed studies on the prevalence and harboring of multiple genes in MDR bacteria have yet been performed in the UAE. Therefore, the current research focuses on the prevalence of class A ESBL (*bla_TEM_*, *bla_SHV_*, and *bla_CTX-M_*) and carbapenemase-encoding genes (*bla_NDM_*, *bla_IMP_*, *bla_OXA-48_*) in Gram-negative bacterial pathogens isolated from various clinical samples collected from the northern region of the UAE.

## 2. Materials and Methods

### 2.1. Ethical Approval

This study was approved by the Institutional Review Board (IRB) of Gulf Medical University, Ajman, UAE with an approval letter dated 14 October 2021, and reference number IRB/COM/STD/101/Oct.2021. The study design, data collection, study population, and protocol adhered to the World Medical Association (WMA) Declaration of Helsinki (DoH-Oct 2013) and to the 2021 Good Clinical Practice guidelines established by the National Drug Abuse Treatment Clinical Trials Network. In addition, ethics and participant information confidentiality were adhered to in the Declaration of Helsinki. Sample data were obtained from PubMed, Scopus, references from relevant articles, and other databases.

### 2.2. Study Design

A laboratory-based experimental study was conducted on various clinical samples such as urine, blood, pus, and sputum collected and received from various hospitals in the Northern Emirates of the UAE including provinces of Ajman, Sharjah, Umm Al Quwain, and Fujairah. The samples were sent to the Department of Microbiology at Thumbay Laboratory, Thumbay University Hospital (TUH), Ajman, UAE.

### 2.3. Data Collection and Clinical Sample Processing

In accordance with the IRB approval, the patients’ clinical samples and other details were collected from Thumbay Laboratory, TUH for the period from 14 October 2021 to 31 July 2022.

### 2.4. Study Populations

All the clinical samples were received and collected from patients attending the inpatient and outpatient departments of the internal medicine and urology departments at TUH, as well as other Thumbay hospitals and clinics in Ajman, Sharjah, and Fujairah, and other private hospitals and clinics in the northern region of the UAE, including Ajman, Sharjah, Fujairah, and Umm Al Quwain, for bacterial culture testing. The samples were directly collected and received from the patients residing in Ajman and Umm Al Quwain by the phlebotomy department and other patient samples were collected/received by the hospitals and clinics in concern situated in other emirates of UAE, and immediately transported using transport media to the phlebotomy department and then sent to the Microbiology department at Thumbay Laboratory, TUH, for culturing bacterial pathogens, staining, and antibiotic sensitivity testing of the isolated bacterial pathogens. The inclusion criteria included all clinical samples received for the purpose of culturing bacteria during the study period. Blood samples specifically designated for viral antigen detection, fungal culture, and other tests such as genetic or biochemical tests not involving bacterial culture tests were excluded.

### 2.5. Bacterial Isolation and Staining

The clinical samples were plated on MacConkey’s agar to isolate bacterial colonies belonging to the Enterobacteriaceae family including *E. coli* and *K. pneumoniae* (fermentative Gram-negative bacteria), CysteineLactose-Electrolyte-Deficient (CLED) agar for urinary tract pathogens, and Cetrimide Agar (MEDYSINAL FZCO, Dubai, United Arab Emirates) for *Pseudomonas* species. Blood agar (MEDYSINAL FZCO, Dubai, United Arab Emirates) was used for the isolation of Gram-positive bacteria and other non-fermentative Gram-negative bacteria. Gram staining of the clinical samples, except for the blood sample, was performed side by side while inoculating the clinical samples on the appropriate culture media plates. All culture plates were incubated overnight at 37 °C for 18–24 h. After isolating bacterial colonies from the different culture media, they were again Gram-stained to confirm whether they are Gram-negative or Gram-positive bacteria [19,20].

### 2.6. Bacterial Identification and Antibiotic Sensitivity Test

MicroScan WalkAway automated detection system was used to identify the isolated bacterial pathogens from the different agar medium and determine their MDR nature. The bacterial inoculum was prepared from single colonies of isolated bacteria using the Prompt™ Inoculation System-D (Beckman Coulter, Brea, CA, USA), which consists of a wand designed to hold a specific quantity of bacteria and a 30 mL Prompt™ Inoculation Water bottle (Beckman Coulter, Brea, CA, USA). The inoculum was thoroughly suspended, poured into a RENOK inoculator tray, and then transferred into the MicroScan Neg Breakpoint Combo 50 panel for Gram-negative organism plus ESβL Panels (B1027-101; Beckman Coulter) [21] using RENOK inoculator (Beckman Coulter) to obtain confirmation and susceptibility testing for EsβL and carbapenemase-producing organisms using Clinical and Laboratory Standards Institute (CLSI) recommended guidelines [22]. The plate was incubated at 16–18 °C in the MicroScan WalkAway automated system DxM 1096 (B1018-496; Beckman Coulter) [23]. Supplementary Tables S1 and S2 demonstrates various tests performed on the MicroScan Neg Breakpoint Combo 50 panel. The Beckman Coulter MicroScan LabPro software WalkAway 9631049 was used to evaluate the Gram-negative bacterial pathogens based on their biochemical utilization and antibiotic sensitivity pattern, especially ESBL and carbapenem-resistant strains [24]. The details of the MicroScan Neg Breakpoint Combo 50 panel for Gram-negative bacteria are provided in the Supplementary Tables S1 and S2.

### 2.7. Detection of ESBL and Carbapenem-Resistant Genes

**Figure 1:**
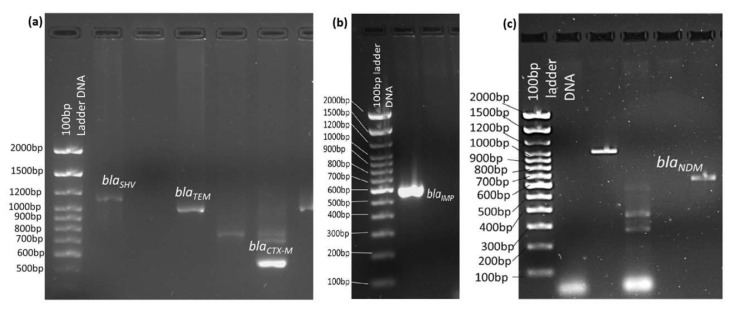
Detection of ESBL genes (**a**) *bla_CTX-M_*, *bla_TEM_*, and *bla_SHV_*, and *carbapenemase genes (***b**) *bla_IMP_*_,_ and (**c**) *bla_NDM_* gene bands and their base pair (bp) sizes using a 100 bp ladder DNA in 1% agarose gel electrophoresis.

After the identification of MDR bacteria, they were sub-cultured in sterile double-strength Muller Hinton broth at 37 °C for 24 h for bacterial DNA extraction using the G-spinTM for Genomic DNA extraction kit (iNtRON Bio, Seongnam-si, Gyeonggi-do, Republic of Korea) [25]. The DNA extracts that were positive for the 16SrRNA gene in PCR assays were processed for the presence of the class A ESBL genes *bla_SHV_*, *bla_CTX-M_*, *bla_TEM_*, and carbapenem-resistant genes *bla_NDM_*, *bla_IMP_*, and *bla_OXA-48_* with consensus primers in a Veriti^TM^ 96-well Thermal cycler (Applied Bio-systems^TM^, Foster City, MA, USA). The PCR assays were performed with the Thermo Scientific PCR Master Mix kit (ThermoFisher SCIENTIFIC, Waltham, MA, USA). The PCR reaction mixture and volume were prepared as follows: 5.0 µL of bacterial DNA was taken in 6 different PCR tubes. 2.5 µL of the forward primer sequence of each resistant gene was added to their appropriately labeled tubes, and likewise, 2.5 µL of the reverse primer sequence of each resistant gene was added to their tubes. 12.5 µL of PCR master mix was added to all the tubes, and using molecular grade water (2.5 µL), the final reaction volume was made up to 25 µL (ThermoFisher SCIENTIFIC, MA, USA). The PCR amplification cycle program was set as follows: 1 cycle of initial denaturation at 95 °C for 5 min, 30 cycles of denaturation at 94 °C for 45 s, primer annealing at 52 °C for 40 s, and extension at 72 °C for 60 s. One cycle of final extension at 72 °C for 10 min, and the PCR product was held at 4 °C [26]. The resistant gene primers and their specifications are given in Table 1. Primers for all the ESBL and carbapenem-resistant genes were synthesized by e-oligos, Gene Link^TM^, Hawthorne, NY, USA. The PCR products underwent agarose gel electrophoresis to separate the DNA fragments and visualize the target genes as shown in Figure 1.

### 2.8. Partial Genomic Sequencing of bla_CTX-M_

Partial gene sequencing of the most frequently isolated ESBL gene *bla_CTX-M_*, mostly from the pathogen *E. coli*, was performed by Sanger sequencing method and the sequenced product was compared with existing gene sequences in GenBank, NCBI. The cladogram of the phylogenetic tree relationship of the *bla_CTX-M_* was constructed using Mega 11 software. It was constructed using the Dice coefficient with a 1% tolerance limit and 1% optimization. Cluster relatedness of collected isolates with ≥70% similarity was considered to indicate an identical pattern type.

### 2.9. Statistical Analysis

Data were analyzed using the Statistical Package for the Social Sciences, version 28.0 for Windows (IBM Corp., Armonk, NY, USA). A chi-square test was conducted to determine the association between different clinical samples (urine, blood, pus, and sputum) and bacterial growth (presence or absence of bacterial growth). The results are presented in Table 2; the significance is indicated at *p* value ≤ 0.05.

## 3. Results

**Figure 2:**
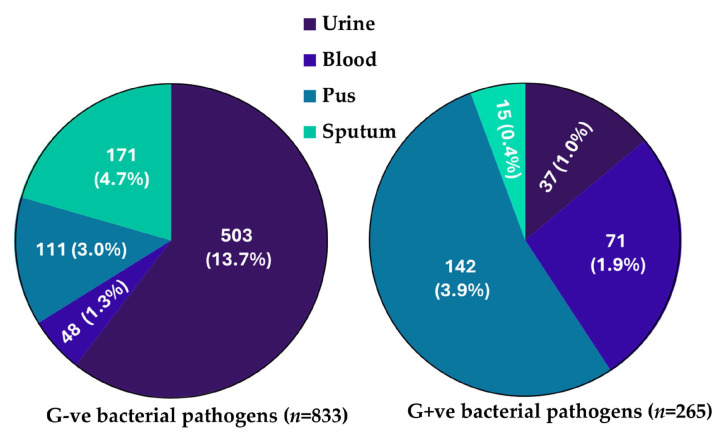
Number of Gram-negative and Gram-positive bacterial pathogens isolated from various clinical samples such as urine, blood, pus, and sputum.

A total of 3670 samples were received, including pus (*n* = 469; 12.8%), blood (*n* = 879; 23.9%), sputum (*n* = 352; 9.6%), and urine (*n* = 1970; 53.7%), and processed for routine bacterial culture. The details are provided in Table 2 and Figure 2.

### 3.1. Isolation of Bacterial Pathogens

In 1098 (30%) clinical samples, bacterial pathogens were isolated and 2572 (70%) showed no growth. The highest positive bacterial growth was detected in the urine samples (*n* = 540; 49%), followed by pus (*n* = 253; 23%), blood (*n* = 119; 11%), and sputum (*n* = 186; 17%). Out of the 1098 positive samples, 833 (76%) were Gram-negative bacterial pathogens, and 265 (24%) were Gram-positive bacteria as shown in Table 2 and Figure 2. The results showed that the pus samples had the highest level of bacterial growth at 61%, followed by sputum samples with a bacterial growth rate of 53%. Urine samples showed a bacterial growth percentage of 27%, while blood samples had the lowest percentage of bacterial growth at 12.6%. The difference between the clinical samples is statistically significant, χ^2^(3, 3670) = 335, *p* < 0.001.

### 3.2. Bacterial Identification and Antibiotic Susceptibility Test

**Figure 3:**
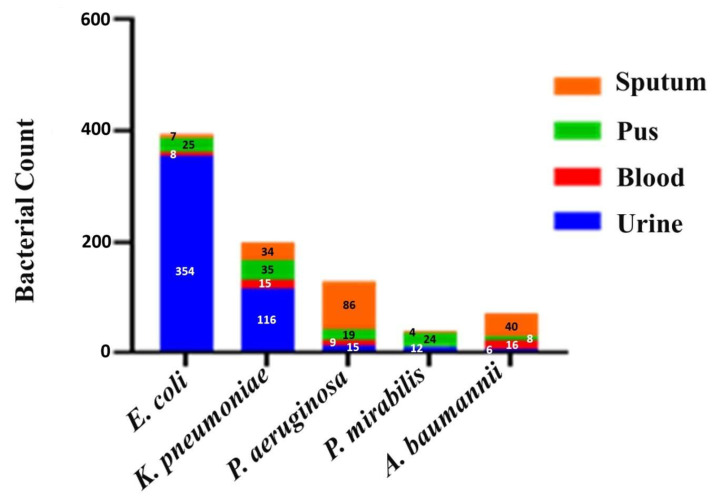
Number of different studied bacterial pathogens isolated from various clinical samples.

Among the Gram-negative bacterial isolates, 249 (30%) were ESBL producers (*n* = 209; 25%) and both ESBL and carbapenemase producers (*n* = 40; 5%). Among the 249 bacterial isolates, 157 (63%) were *E. coli*, 45 (18%) were *K. pneumoniae*, 20 (8%) were *P. aeruginosa*, 17 (7%) were *Proteus mirabilis*, and 10 (4%) were *A. baumannii*, as shown in Table 3; Figure 3.

**Figure 4:**
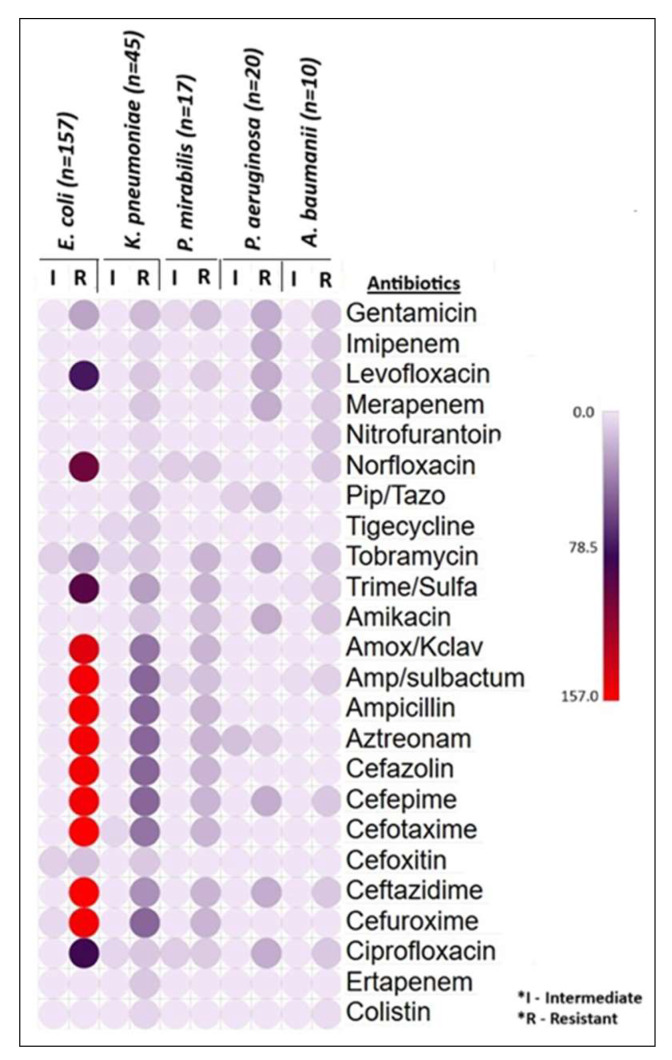
Number of intermediate and resistant patterns of *E. coli*, *K. pneumonia*, *P. mirabilis*, *P. aeruginosa*, and *A. baumannii* to various common antibiotics.

Among the ESBL-producing *E. coli* isolates (*n* = 157), 100% resistance was observed against cefotaxime and ceftazidime. High to moderate resistance rates were recorded for ampicillin/sulbactam, ampicillin, aztreonam, cefazolin, cefepime, cefuroxime (97.5%), amoxicillin/clavulanate (92.5%), norfloxacin (63.7%), trimethoprim/sulfamethoxazole (58.6%), ciprofloxacin (51.6%), and levofloxacin (46.5%). Lower resistance rates were noted for gentamicin (14.6%) and tobramycin (12.7%), while the lowest resistance was observed for cefoxitin (7.6%). Notably, all the *E. coli* isolates were 100% sensitive to other remaining antibiotics. In *K. pneumoniae* isolates (*n* = 45), 100% resistance was observed in ampicillin, ampicillin/sulbactam, aztreonam, cefazolin, cefepime, and cefuroxime. High resistance rates were also noted for amoxicillin/clavulanate (89%) and cefotaxime (89%). Moderate resistance levels were found for ceftazidime (66.7%), nitrofurantoin (66.7%), norfloxacin (55.6%), and trimethoprim/sulfamethoxazole (55.6%). Variable resistance were noted to multiple antibiotics and no complete susceptibility observed on the *K. pneumoniae* isolates. For ESBL-producing *P. mirabilis* isolates (*n* = 17), 100% resistance was observed to 10 antibiotics including beta-lactams and tobramycin. 76.5% of *P. mirabilis* isolates resistance to gentamicin and amikacin, 52.9% of isolates were resistant to norfloxacin, ciprofloxacin, and levofloxacin. The bacteria showed intermediate resistance to norfloxacin, ciprofloxacin, gentamicin, and ampicillin/sulbactam. They were 100% sensitive to the remaining antibiotics. *P. aeruginosa* (*n* = 20) showed 100% resistance to the drugs gentamicin, imipenem, levofloxacin, meropenem, tobramycin, amikacin, cefepime, ceftazidime, and ciprofloxacin. 65% and 35% of them were resistant to the drugs piperacillin/tazobactam and aztreonam, respectively. None of them were 100% sensitive to any antibiotics used in the test except colistin. Similarly, the bacteria *A. baumannii* (*n* = 10) showed 100% resistance to 11 different drugs. 70–80% of the strains were resistant to ampicillin/sulbactam and trimethoprim/sulfamethoxazole, and 30% of them were resistant to colistin. The details of the antibiotic sensitivity pattern of all the isolates are illustrated in Table 4 and Figure 4.

### 3.3. Unveiling the ESBL and Carbapenem-Resistant Genes in Gram-Negative Bacterial Pathogens

**Figure 5:**
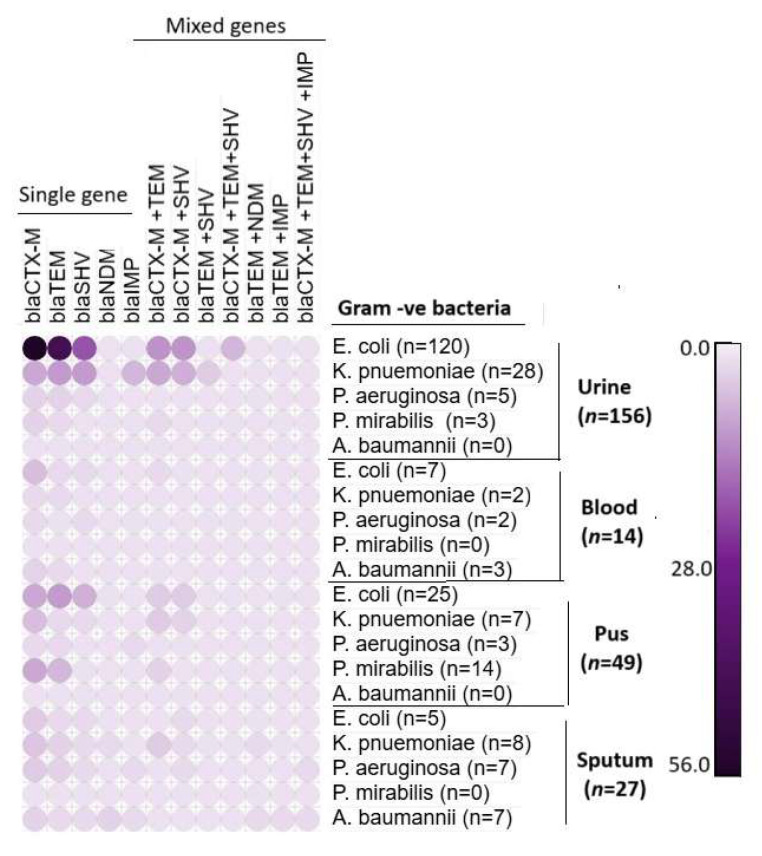
Frequency of single and mixed resistant ESBL and carbapenemase-encoding genes detected in *E. coli*, *K. pneumoniae*, *P. aeruginosa*, *P. mirabilis*, and *A. baumannii*.

All the ESBL and carbapenem-resistant bacteria (*n* = 249) had positive results for all three ESBL genes and carbapenem-resistant genes *bla_NDM_* and *bla_IMP_*, either harboring them singly or in multiple copies. Among the 249 isolates, *E. coli* (*n* = 157; 63%) harbored all the ESBL genes, with *bla_CTX-M_* (*n* = 72; 46%) being the most identified, followed by *bla_TEM_* (*n* = 59; 37.6%) and *bla_SHV_* (*n* = 31; 20%). Of the 157 *E. coli* isolates, 20.33% (*n* = 32) co-harbored more than one gene, such as *bla_CTX-M+TEM_* or *bla_CTX-M+SHV_*. In these combinations, *bla_CTX-M+TEM_* (*n* = 15; 9.5%) and *bla_CTX-M+SHV_* (*n* = 15; 9.5%) were detected in 15 *E. coli* strains, while all three genes were identified together in 3.8% of the *E. coli* isolates. Similarly, *bla_CTX-M_* (*n* = 18; 40%) was the predominant gene in MDR *K. pneumoniae* (*n* = 45), followed by *bla_TEM_* (*n* = 14; 31%), *bla_SHV_* (*n* = 14; 31%), *bla_IMP_* (*n* = 6; 13.3%), and *bla_NDM_* (*n* = 1; 2.2%). Combinations of genes like *bla_CTX-M+TEM_* (*n* = 14; 31%), *bla_CTX-M+SHV_* (*n* = 10; 22%), *bla_TEM+SHV_* (*n* = 3; 6.6%), or *bla_TEM+NDM_* (*n* = 1; 2%) were detected in 62.22% of the *K. pneumoniae* isolates. In *P. aeruginosa*, *bla_CTX-M_* and *bla_TEM_* were detected in almost all isolates, with *bla_IMP_* found in 7 (35%) strains and *bla_SHV_* in 13 (65%) strains. Gene combinations like *bla_CTX-M+SHV_* (*n* = 1; 5%) and *bla_CTX-M+TEM+SHV+IMP_* (*n* = 1; 5%) were detected in 10% of the organisms. Similarly, *bla_CTX-M_* was detected in all 17 ESBL-positive *P. mirabilis* strains (100%), with *bla_TEM_* found in 8 strains (47.1%). Among the 17 *P. mirabilis* strains, 17.6% (*n* = 3) were positive for both genes (*bla_CTX-M+TEM_*). These resistant-gene-carrying bacterial strains were predominantly isolated from urine and pus samples, as detailed in Table 5 and Figure 5.

**Figure 6:**
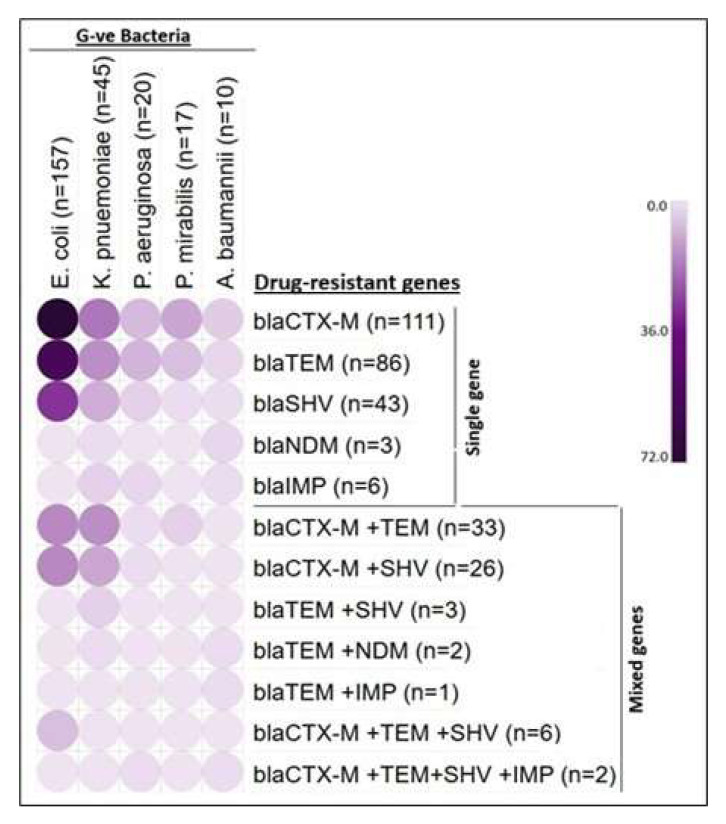
Frequency of detection of ESBL and carbapenemase-resistant genes in the studied bacterial pathogens.

All the *A. baumannii* isolates *(n* = 10; 100%) showed positive for the genes *bla_CTX-M_* and *bla_TEM_*. Additionally, 20% (*n* = 2) of the strains individually carried the genes *bla_NDM_* and *bla_SHV_*, while *bla_IMP_* was detected in 10% of the *A. baumannii* isolates from the sputum samples. The gene combinations *bla_TEM+NDM_*, *bla_TEM+IMP_*, and *bla_CTX-M+TEM+SHV+IMP_* were each detected in 10% (*n* = 1) of the strains. None of the ESBL and carbapenem-producing strains tested positive for the gene *bla_OXA-48_* and the above-mentioned details are given in Table 6 and Figure 6.

### 3.4. Genomic Sequencing and Phylogenetic Tree Relationships of the bla_CTX-M_

**Figure 7:**
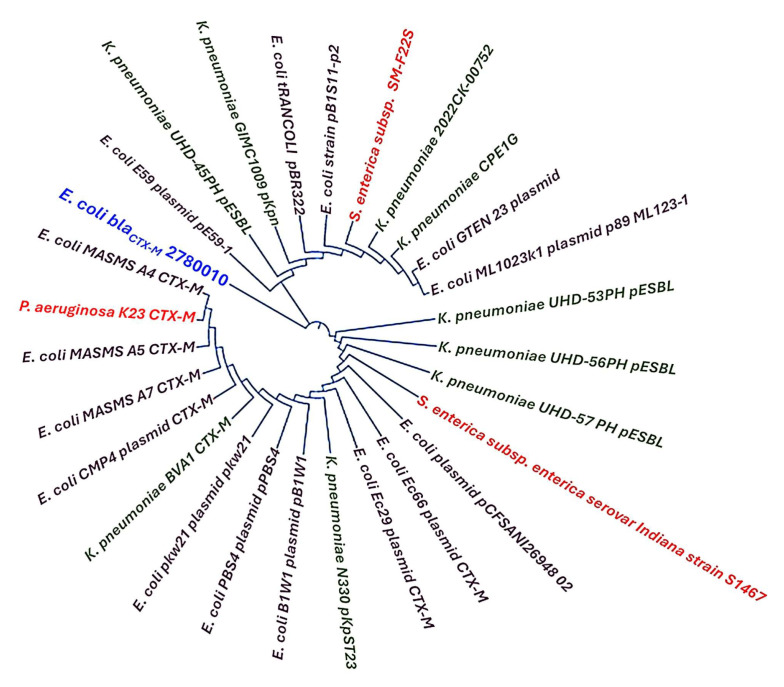
Cladogram of the phylogenetic tree of the gene *bla_CTX-M_* illustrates a 99.74% similarity with other *bla_CTX-M_* genes from various *E. coli* and *K. pneumoniae* isolates. The software Mega 11 was used to construct the tree.

The partial genomic sequence of the gene *bla_CTX-M_* was compared with other existing *bla_CTX-M_* genes from various Gram-negative bacterial pathogens already deposited in the GenBank, NCBI. Our genomic sequence *bla_CTX-M_* showed 99.74% similarity with *bla_CTX-M_* genes from *E. coli* and *K. pneumoniae.* Using the 99.74% similarity index, a cladogram of the phylogenetic tree was constructed. The cladogram of the phylogenetic tree is illustrated in Figure 7.

## 4. Discussion

The present study collected 3670 various clinical samples, of which the urine samples were predominantly (51.2%) received and had shown the highest bacterial growth rate (49.1%). The bacterial growth rate was significant among the samples collected, and the overall positivity rate of the bacterial growth was 30%.

Over the recent decades, the spread of ESBL and carbapenemase-enzyme-producing Enterobacteriaceae and non-fermentative Gram-negative bacteria, especially *P. aeruginosa*, *P. mirabilis*, and *A. baumannii*, pose a significant threat to healthcare of Arabian Peninsula region [28]. These organisms exhibit resistance to multiple classes of antibiotics, including last-resort drugs like carbapenems, limiting treatment options and increasing morbidity, mortality, and healthcare costs [14,29]. Reduced treatment options, complex infections, and costly treatments are some of the major concerns for people infected with ESBL and carbapenemase-enzyme-producing organisms. A major driver of ESBL and carbapenemase-producing Gram-negative fermentative and non-fermentative bacteria is the horizontal transfer of mobile genetic elements carrying genes for ESBL and/or carbapenemase enzyme production [13].

In this study, 64.3% of the bacterial isolates were Gram-negative bacteria and the remaining 35.7% were Gram-positive bacteria. Among the Gram-negative bacteria, the *E. coli* (42.3%) was the most predominant isolate followed by *K. pneumoniae* (19.4%), *P. aeruginosa* (13.5%), *A. baumannii* (7.3%), and *P. mirabilis* (3.3%). In 2020, National AMR Surveillance system of the UAE [30] reported that the *E. coli* was the most commonly (80%) isolated Gram-negative bacteria, then *K. pneumoniae* (55–60%), *P. aeruginosa* (20%), *A. baumannii* (<10%), and *P. mirabilis* (<5%).

A comparison of the resistance rate of ESBL *E. coli* strains to previous studies across other Gulf Cooperation Council (GCC) countries revealed similar patterns. With 100% resistance to drugs such as cefotaxime and ceftazidime [27,31], there was a 46.5–97.5% (medium–high resistance) rate to 11 different beta-lactams and a low resistance rate to gentamycin and tobramycin [32,33]. Furthermore, our study found ESBL *E. coli* showing 100% sensitivity to eight antibiotics (Table 4) which is similar to studies from Qatar and Saudi Arabia [34,35]. The predominance of ESBL *E. coli* in urine samples (Table 5) highlights the widespread occurrence of urinary tract infections (UTIs). UTIs are common, but when caused by ESBL strains, they can lead to severe complications such as sepsis, prolonged hospital stays, and even increased mortality [36].

The ESBL *K. pnuemoniae* strains showed complete resistance to various antibiotics like ampicillin, aztreonam, cefazolin, cefepime, and cefuroxime which was consistent with findings from studies conducted in UAE [37,38] and other GCC countries [15,31,34,39]. The strain exhibited 89% resistance rate to cefotaxime and amoxicillin + clavulanate but in contrast, a lower resistance rate to the same drugs was reported in Kuwait [40], UAE [38], Qatar [34], and Saudi Arabia [32]. Interestingly, the rate of resistance to antibiotics such as tigecycline and meropenem in our study rose to 22% from a mere 8% reported in previous studies indicating the prevalence of carbapenem-resistant *K. pnuemoniae* [27,41]. Moreover, there has been a significant rise in the colistin resistance rate from 0 to over 20% over the last decade, posing a serious threat as colistin is currently the last-line treatment for carbapenem-resistant *K. pneumoniae* [28]. Antibiotic sensitivity pattern of ESBL *P. mirabilis* revealed high resistance to most beta-lactams as expected but least resistance to carbapenems with sensitivity to colistin. Similarly, a study in Saudi Arabia reported *P. mirabilis* being susceptible to carbapenems such as meropenem, imipenem, etc., but was reported as intrinsically resistant to colistin [42]. *P. mirabilis* is the second leading cause of UTIs, after *E. coli*, hence using carbapenems or piperacillin/tazobactam antibiotics can help manage ESBL *P. mirabilis* infections effectively.

The bacteria *P. aeruginosa* was 100% resistant to nine different drugs and 100% susceptible to colistin and medium–low resistance rate was noted in piperacillin/tazobactam (65%) and aztreonam (35%). In contrast to our study, the studies from Sudan [43] and Egypt [44] observed that the bacteria had low resistance rate and was highly susceptible to the above-mentioned drugs. In the case of *A. baumannii*, the bacteria were 100% resistant to 11 drugs (Table 4), 70–80% resistance rate was observed with ampicillin/sulbactam and trimethoprim/sulfamethoxazole and a 30% resistance to colistin. This was higher compared to studies from Oman [45] and Iran [46]. Resistance to colistin is rapidly emerging among the MDR *A. baumannii* and *K. pneumoniae*, which is becoming a global burden [47,48].

Molecular characterization of the resistant Gram-negative isolate was carried out to determine prevalence rate of class A ESBL genes (*bla_CTX-M_*, *bla_SHV_*, *bla_TEM_*) and carbapenem-resistant genes (*bla_IMP_*, *bla_NDM_*, *bla_0XA-48_*) within UAE. The most predominant ESBL gene identified was *bla_CTX-M_* with high prevalence in *E. coli* (45.9%), *P. mirabilis* (58.8%), *K. pneumoniae* (42.2%), *A. baumannii* (40%), and *P. aeruginosa* (35%) followed by *bla_TEM_* and *bla_SHV_* mainly in the *E. coli* (35.6% and 18.5%) and *K. pneumoniae* (16.4% and 26.7%). A similar pattern of gene prevalence was noted in other GCC countries [27,34,38,39]. In contrast, studies from Iraq [49], India [50], and Egypt [51] stated that the *bla_TEM_* was the most predominant gene in the *Enterobacterales* followed by *bla_CTX-M_* and *bla_SHV_*.

The carbapenemase-encoding gene *bla_IMP_* was detected in 13.3% of *K. pneumoniae* isolated from the urine samples and in 10% of *A. baumannii* from sputum and 10% of *P. aeruginosa* from both the samples. Likewise, 20% of *A. baumannii* and 2.2% of *K. pneumoniae* harbored the *bla_NDM_*. Almost 94% of them were positive to the *bla_IMP_* in Bharain [52] where 6% of the *A. baumannii* were positive to the *bla_NDM_*, whereas 8.7% of the *K. pneumoniae* were positive to the *bla_IMP_* in Saudi Arabia [41]. A global perspective on carbapenem-resistant *A. baumannii* (CRAB) revealed predominance of *bla_OXA-48_* [53]. In neighboring countries like Saudi Arabia, roughly 71% of the carbapenem-resistance gene was OXA-48, mostly detected on *K. pneumoniae* CRE isolates [41]. In the North African country Morocco, 83% of carbapenemase-producing Enterobacterales carried OXA-48 [54]. However, a study from Qatar stated that 30% of the carbapenemase producers carried OXA-48 gene and NDM-1 as well [34]. But in contrast, our study did not find *bla_OXA-48_* amongst any bacterial isolate. The emergence of carbapenem-resistant bacterial isolates poses a significant threat, as carbapenems are considered the last-resort antibiotics for treating infections caused by ESBL-producing pathogens.

Additionally, our study found co-existence of resistant genes in various combinations in bacterial pathogens. The highest co-existence patterns were *bla_CTX-M+TEM_* and *bla_CTX-M+SHV_* represented by *E. coli* (9.6% of each), *K. pneumoniae* (31.1% and 22.2%), and *P. aeruginosa* (15% of each). In 17.65% of the *P. mirabilis*, the co-existence gene was *bla_CTX-M+TEM_*. The lowest co-existence patterns were *bla_TEM+SHV_* and *bla_CTX-M+TEM+SHV_* represented by *K. pneumoniae* (6.67%) and *E. coli* (3.82%), respectively. Similarly, the highest co-existence of the gene *bla_CTX_-_M+TEM_* (21%) was found in the ESBL *E. coli and K. pneumoniae* from Qatar [34], Saudi Arabia [15,27], and followed by the least co-existence of the genes *bla_TEM+SHV_*, *bla_CTX-M+SHV_*, and *bla_CTX-_*_M+TEM+SHV_ which were detected in the same studies; in contrast, Moglad et al. [55], Badger-Emeka et al. [29], and Sid-Ahmed et al. [56] found that the highest co-harbored genes were *bla_CTX-M+TEM+SHV_* (50% and 24.7%) and *bla_CTX-M+SHV_* (50% and 10.1%) found in the *E. coli*, *K. pneumoniae*, and other *Enterobacterales* in their studies. Likewise, the lowest co-expression of the genes *bla_TEM+NDM_*, *bla_TEM+IMP_*, and *bla_CTX-M+TEM+SHV+IMP_* were also detected in 2.22% *K. pneumoniae*, 5% *P. aeruginosa,* and 10% of each *A. baumannii* to each gene combination. In such a way, Elbadawi HS et al. found in their study that the lowest co-expression of the ESBL and carbapenemase-producing genes were noticed in the *K. pneumoniae*, *A. baumannii*, *E. coli*, and *P. aeruginosa* [57].

As with all other studies, this study also reported a few limitations as follows. The study samples were received from TUH, other Thumbay hospitals, and clinics located in and around the northern emirates of the UAE (Ajman, Sharjah, and Fujairah) as well as other hospitals and clinics from the same northern part of the UAE, including Umm Al Quwain. However, the study samples were not received and collected from other emirates including Dubai, Abu Dhabi, and Ras Al Khaimah. The carbapenemase-encoding gene *bla_OXA-48_* was not detected in any of the ESBL and carbapenem-resistant Gram-negative bacterial pathogens, even in *A. baumannii*. In this study, genomic sequencing of the predominant gene *bla_CTX-M_* was performed by the Sanger method, but no other class A genes were sequenced. In addition, patient demographic details and their clinical correlations, including critical care unit patients, are not included in this study and statistical analysis correlating genotypic profiles with phenotypic resistance patterns was not conducted. These important limitations should be addressed in future studies.

## 5. Conclusions

The current findings confirm UAE to have a slightly high prevalence of the ESBL and carbapenem-resistant genes. According to the study the gene *bla_CTX-M_*, *bla_TEM_* and *bla_SHV_* were the most detected in the *E. coli* and *K. pneumoniae* especially isolated from the urine samples. The highest percentage of the bacteria *K. pneumoniae* harbored all the ESBL and carbapenemase-resistant genes, and the multiple two-gene combinations like *bla_CTX-M+TEM_*, *bla_CTX-M+SHV_*, *bla_TEM+SHV_*, and *bla_TEM+NDM_* as well. *A. baumannii* also harbored all the ESBL and carbapenem-resistant genes, and co-harbored the genes *bla_TEM+NDM_*, *bla_TEM+IMP_*, and *bla_CTX-M+TEM+SHV+IMP_*. The current findings underscore the critical importance of rational antibiotic therapy, supported by ongoing surveillance and robust infection control strategies, to effectively curb the spread of ESBL and carbapenem-resistant genes in Gram-negative bacteria. In the UAE, where *bla_CTX-M_*_-_mediated resistance is particularly dominant, empirical antibiotic therapy must be strategically revised based on local antimicrobial resistance patterns. A multifaceted approach combining data-driven antibiotic selection, strong antimicrobial stewardship, enhanced molecular surveillance of resistance genes, and enhanced infection prevention measures is essential to manage and contain the rising threat of multidrug-resistant ESBL-producing pathogens in the region.

## Figures and Tables

**Table 1:** Details of the primer sequences, amplicon size, and annealing temperature of the ESBL and Carbapenem-resistant genes.

Gene Type	Amplicon	Primer Sequence (5′-3′)	Amplicon Size (bp)	Annealing Temperature °C	Reference
ESBL gene primers	*bla_CTX-M_*	F-ATGTGCAGYACCAGTAARGTKAT GCC R-TGGGTRARRTARGTSACCAGAAY CAGCGG	593	58	[15]
	*bla_TEM_*	F-TCGCCGCATACACTATTCTCAGAA TGA R-ACGCTCACCGGCTCCAGATTTAT	445	50	
	*bla_SHV_*	F-GGGTTATTCTT ATTTGTCGC, R-TTAGCGTTGCCAGTGCTC	747	58	
Carbapenem-resistant gene primers	*bla_NDM_*	F-GGTTTG GCGATCTGGTTTTC R-CGGAATGGCTCATCACGATC	621	57	[27]
	*bla_IMP_*	F-CTACCGCAGCAGAGTCTTTG R-AACCAGTTTTGCCTTACCAT	587	56	
	*bla*_OXA48_	F-GCGTGGTTAAGGATGAACAC R-CATCAAGTTCAACCCAACCG	438	57	[15]
Control primer	16S rRNA gene	F-AGAGTTTGATCMTGGCTCAG R-ACGGHTACCTTGTTACGACTT	1500	55	

**Table 2:** No. of various clinical samples for positive bacterial growth.

Clinical Samples *n* = 3670 (%)	No. of Bacterial Pathogens Isolated *n* = 1098 (29.9%)		No. of Samples for No Bacterial Growth *n* = 2572 (70.1%)	*p*-Value/ Chi-Square Test
	Gram-Negative Bacteria *n* = 833 (22.7%)	Gram-Positive Bacteria *n* = 265 (7.2%)		
Urine *n* = 1970 (53.7%)	503 (13.7%)	37 (1.0%)	1430 (39%)	<0.001/335 *
Blood *n* = 879 (23.9%)	48 (1.3%)	71 (1.9%)	760 (20.7%)	
Pus *n* = 469 (12.8%)	111 (3.0%)	142 (3.9%)	216 (5.9%)	
Sputum *n* = 352 (9.6%)	171 (4.7%)	15 (0.4%)	166 (4.5%)	

* Chi-square test.

**Table 3:** Total no. of studied bacteria is isolated from various clinical samples.

No. of Samples Positive for Gram-Ve Bacteria *n* (%)	No. of Gram-Ve Bacteria Isolated *n* = 833				
	*E. coli* *n* = 394 (47.2%)	*K. pneumoniae* *n* = 200 (24%)	*P. aeruginosa* *n* = 129 (15.5%)	*P. mirabilis* *n* = 40 (4.8%)	*A. baumannii* *n* = 70 (8.4%)
Urine *n* = 503 (60.3%)	354 (42.5%)	116 (13.9%)	15 (1.8%)	12 (1.4%)	6 (0.7%)
Blood *n* = 48 (5.7%)	8 (0.9%)	15 (1.8%)	9 (1.1%)	0	16 (1.9%)
Pus *n* = 111 (13.3%)	25 (3.0%)	35 (4.2%)	19 (2.3%)	24 (2.9%)	8 (1.0%)
Sputum *n* = 171 (20.5%)	7 (0.8%)	34 (4.1%)	86 (10.3%)	4 (0.5%)	40 (4.8%)

**Table 4:** Antibiotic sensitivity pattern of the studied bacteria isolated from various clinical samples.

Antibiotics	*E. coli* *n* = 157 (%)		*K. pneumoniae* *n* = 45 (%)		*P. mirabilis* *n* = 17 (%)		*P. aeruginosa* *n* = 20 (%)		*A. baumannii* *n* = 10 (%)	
	I	R	I	R	I	R	I	R	I	R
Gentamicin	0	23 (15%)	0	15 (33%)	4 (23%)	13 (77%)	0	20 (100%)	0	10 (100%)
Imipenem	0	0	0	5 (11%)	0	0	0	20 (100%)	0	10 (100%)
Levofloxacin	0	73 (46%)	0	10 (22%)	0	8 (47%)	0	20 (100%)	0	10 (100%)
Meropenem	0	0	0	10 (22%)	0	0	0	20 (100%)	0	10 (100%)
Nitrofurantoin	0	0	0	5 (11%)	0	0	0	0	0	10 (100%)
Norfloxacin	0	100 (64%)	0	5 (11%)	8 (47%)	9 (53%)	0	0	0	10 (100%)
Piperacillin/Tazobactam	0	0	0	10 (22%)	0	0	7 (35%)	13 (65%)	0	0
Tigecycline	0	0	5 (11%)	10 (22%)	0	0	0	0	0	0
Tobramycin	7 (5%)	20 (13%)	5 (11%)	10 (22%)	0	17 (100%)	0	20 (100%)	0	10 (100%)
Trimethoprim/ Sulfamethoxazole	0	92 (59%)	0	25 (55%)	0	17 (100%)	0	0	2 (20%)	8 (80%)
Amikacin	0	0	0	10 (22%)	0	13 (77%)	0	20 (100%)	0	10 (100%)
Amoxicillin + Clavulanate	0	145 (92%)	0	40 (89%)	0	17 (100%)	0	0	0	0
Ampicillin/ sulbactam	0	153 (97%)	0	45 (100%)	4 (23%)	13 (77%)	0	0	3 (30%)	7 (70%)
Ampicillin	0	153 (97%)	0	45 (100%)	0	17 (100%)	0	0	0	0
Aztreonam	0	153 (97%)	0	31 (69%)	0	17 (100%)	13 (65%)	7 (35%)	0	0
Cefazolin	0	153 (97%)	0	45 (100%)	0	17 (100%)	0	0	0	0
Cefepime	0	153 (97%)	0	45 (100%)	0	17 (100%)	0	20 (100%)	0	10 (100%)
Cefotaxime	0	157 (100%)	5 (11%)	40 (89%)	0	17 (100%)	0	0	0	0
Cefoxitin	7 (5%)	12 (7.6%)	0	10 (22%)	0	0	0	0	0	0
Ceftazidime	0	157 (100%)	0	30 (67%)	0	17 (100%)	0	20 (100%)	0	10 (100%)
Cefuroxime	4 (3%)	153 (97%)	0	45 (100%)	0	17 (100%)	0	0	0	0
Ciprofloxacin	0	81 (52%)	5 (11%)	10 (22%)	8 (47%)	9 (53%)	0	20 (100%)	0	10 (100%)
Ertapenem	0	0	0	10 (22%)	0	0	0	0	0	0
Colistin	0	0	0	5 (11%)	0	0	0	0	0	3 (30%)

**Table 5:** Number of ESBL and carbapenem-resistant genes in number of *E. coli*, *K. pneumoniae*, *P. aeruginosa*, *P. mirabilis*, and *A. baumannii* isolated from various clinical samples.

No. of Resistant Bacteria in Clinical Samples *n* = 249 (%)		Single Gene Detection					Multiple Gene Detection						
		*bla_CTX-M_* *n* = 111	*bla_TEM_* *n* = 85	*bla_SHV_* *n* = 45	*bla_NDM_* *n* = 3	*bla_IMP_* *n* = 6	*bla_CTX-M_* *_+TEM_* *n* = 33	*bla_CTX-M_* *_+SHV_* *n* = 26	*bla_TEM_* *_+SHV_* *n* = 3	*bla_CTX-M+TEM +SHV_* *n* = 6	*bla_TEM +NDM_* *n* = 2	*bla_TEM_* *_+IMP_* *n* = 1	*bla_CTX-M+TEM+SHV+IMP_* *n* = 2
**Urine** *n* = *156*	*E. coli* *n* = 120 (77%)	56 (46.7%)	44 (36.7%)	20 (16.7%)	0	0	11 (9%)	11 (9%)	0	6 (5%)	0	0	0
	*K. pnuemoniae* *n* = 28 (18%)	8 (28.6%)	10 (35.7%)	7 (35.7%)	0	3 (21.4%)	8 (28.6%)	7 (25%)	3 (10.7%)	0	0	0	0
	*P. aeruginosa* *n* = 5 (3%)	2 (40%)	2 (40%)	1 (20%)	0	0	0	0	0	0	0	0	0
	*P. mirabilis* *n* = 3 (2%)	2 (66%)	1 (33%)	0	0	0	1 (33%)	0	0	0	0	0	0
	*A. baumannii* *n* = 0	0	0	0	0	0	0	0	0	0	0	0	0
**Blood** *n* = *14*	*E. coli* *n* = 7 (50%)	5 (71.4%)	1 (14.3%)	1 (14.3%)	0	0	1 (14.3%)	0	0	0	0	0	0
	*K. pnuemoniae* *n* = 2 (14.3%)	1 (50%)	1 (50%)	0	0	0	0	0	0	0	0	0	0
	*P. aeruginosa* *n* = 2 (14.3%)	1 (50%)	0	1 (50%)	0	0	0	0	0	0	0	0	0
	*P. mirabilis* *n* = 0	0	0	0	0	0	0	0	0	0	0	0	0
	*A. baumannii* *n* = 3 (2%)	2 (66%)	1 (33.3%)	0	0	0	0	0	0	0	0	0	0
**Pus** *n* = *49*	*E. coli* *n* = 25 (51%)	8 (32%)	10(40%)	7 (28%)	0	0	3 (12%)	3 (12%)	0	0	0	0	0
	*K. pnuemoniae* *n* = 7 (14.3%)	5 (71.4%)	1 (14.3%)	1 (14.3%)	0	0	3 (43%)	2 (28.6%)	0	0	0	0	0
	*P. aeruginosa* *n* = 3 (6%)	1 (33%)	1 (33%)	0	0	1 (33%)	0	0	0	0	0	0	0
	*P. mirabilis* *n* = 14 (28.6%)	8 (57%)	4 (43%)	0	0	0	2 (14.3%)	0	0	0	0	0	0
	*A. baumannii* *n* = 0	0	0	0	0	0	0	0	0	0	0	0	0
**Sputum** *n* = *30*	*E. coli* *n* = 5 (16.7%)	3 (60%)	1 (20%)	1 (20%)	0	0	0	1 (20%)	0	0	0	0	0
	*K. pnuemoniae* *n* = 8 (26.6%)	4 (50%)	2 (25%)	1 (12.5%)	1 (12.5%)	0	3 (37.5%)	1 (12.5%)	0	0	1 (12.5%)	0	0
	*P. aeruginosa* *n* = 10 (33%)	3 (42.9%)	5 (28.6%)	1 (14.3%)	0	1 (14.3%)	1 (14.3%)	1 (14.3%)	0	0	0	0	1 (14.3%)
	*P. mirabilis* *n* = 0	0	0	0	0	0	0	0	0	0	0	0	0
	*A. baumannii* *n* = 7 (23%)	2 (28.6%)	1 (14.3%)	1 (14.3%)	2 (28.6%)	1 (14.3%)	0	0	0	0	1 (14.3%)	1 (14.3%)	1 (14.3%)

**Table 6:** Frequency of detection of ESBL and carbapenemase-encoding genes in the studied bacterial pathogens.

Detected Genes		No. of Bacteria Carrying the Resistant Genes *n* = 249				
		*E. coli* *n* = 157 (63%)	*K. pnuemoniae* *n* = 45 (18%)	*P. aeruginosa* *n* = 20 (8%)	*P. mirabilis* *n* = 17 (6.8%)	*A. baumannii* *n* = 10 (4%)
**Single gene**	*bla_CTX-M_ n* = 111	72 (29%)	18 (40%)	7 (35%)	10 (59%)	4 (40%)
	*bla_TEM_ n* = 86	56 (35.7%)	14 (31%)	8 (40%)	6 (35.3%)	2 (20%)
	*bla_SHV_ n* = 43	29 (18.5%)	9 (20%)	3 (15%)	1 (5.9%)	1 (10%)
	*bla_NDM_ n* = 3	0	1 (2.2%)	0	0	2 (20%)
	*bla_IMP_ n* = 6	0	3 (6.6%)	2 (10%)	0	1 (10%)
**Mixed genes**	*bla_CTX-M+TEM_ n* = 33	15 (9.5%)	14 (31%)	1 (5%)	3 (17.7%)	0
	*bla_CTX-M+SHV_ n* = 26	15 (9.5%)	10 (22.2%)	1 (5%)	0	0
	*bla_TEM+SHV_ n* = 3	0	3 (6.6%)	0	0	0
	*bla_TEM+NDM_ n* = 2	0	1 (2.2%)	0	0	1 (10%)
	*bla_TEM+IMP_ n* = 1	0	0	0	0	1 (10%)
	*bla_CTX-M+TEM+SHV_ n* = 6	6 (4%)		0	0	0
	*bla_CTX-M+TEM+SHV+IMP_ n* = 2	0	0	1 (5%)	0	1 (10%)

## Data Availability

The raw data supporting the conclusions of this article will be made available by the authors on request.

## Supplementary Materials

The following supporting information can be downloaded at: https://www.mdpi.com/article/10.3390/microorganisms13081880/s1. Table S1: Test wells on the MicroScan Gram-negative panels (Neg Breakpoint Combo 50); Table S2: Antibiotic sensitivity test wells on the MicroScan Gram-negative panels (Neg Breakpoint Combo 50).

## Abbreviations

The following abbreviations are used in this manuscript:

AMR	Antimicrobial resistance
MDR	Multidrug resistant
ESBL	Extended Spectrum of Beta-lactamase
GMU	Gulf Medical University
TUH	Thumbay University Hospital
GCC	Gulf Cooperation Council
UAE	United Arab Emirates
