# Supplementary material for: Molecular Surveillance of ESBL and Carbapenemase Genes in Gram-Negative Bacterial Pathogens Isolated from Various Clinical Samples Collected from Northern Region of United Arab Emirates
Source: Microorganisms. 2025 Aug 12;13(8):1880. doi: 10.3390/microorganisms13081880 (PMC12388057; doi:10.3390/microorganisms13081880)
Supplement: Supplementary file 1 [file microorganisms-13-01880-s001.zip › microorganisms-3776544-supplementary.pdf]

**Supplementary Table S1.** Test wells on the MicroScan Gram-negative panels (Neg Breakpoint Combo 50). Underlined substrates are covered with mineral oil.

| Abbreviation | Substrate | Abbreviation | Substrate | Abbreviation | Substrate |
|---|---|---|---|---|---|
| GLU | Glucose | LYS | Lysine | OF/B | Oxidation Base control |
| SUC | Sucrose | ARG | Arginine | OF/G | Oxidation of Glucose |
| SOR | Sorbitol | ORN | Ornithine | DCB | Decarboxylase Base control |
| RAF | Raffinose | TDA | Tryptophan diaminase | NIT | Nitrate |
| RHA | Rhamnose | ESC | Esculine | K4 | Kanamycin 4μg/ml |
| ARA | ARabinose | VP | Voges-Praskauer | Cl4 | Colistin 4μg/ml |
| INO | Inocitol | CIT | Citrate | P4 | Penicillin G4μg/ml |
| ADO | Adonitol | MAL | Malonate | Fd64 | Nitrofurantoin 64g/ml |
| MEL | Melibiose | ONPG | O-nitrophenyl -D-Galacto-pyranoside | Cf8 | Cephalotin 8 μg/ml |
| URE | Urea | TAR | Tartrate | To4 | Tobramycin 4 g/ml |
| $H_2S$ | Hydrogen Sulfide | ACE | Acetamide | - | - |
| IND | Indole | CET | Cetrimide | - | - |

**Supplimentary Table S2.** Antibiotic sensitivity test wells on the MicroScan Gram-negative panels (Neg Breakpoint Combo 50).

| **Abbreviation** | **Substrate** | **Concentration (μg/ml)** | **Abbreviation** | **Substrate** | **Concentration (μg/ml)** |
|---|---|---|---|---|---|
| Ak | Amikacin | 16 -32 | Cl | Colistin | 2 -4 |
| Aug | Augmentin | 8/4 – 16/8 | Etp | Ertapenem | 0.5-1 |
| Am | AmiKacin | 8-16 | Gm | Gentamicin | 4-8 |
| A/S | Ampicillin/ Sulbactum | 8/4-16/8 | Imp | Imipenem | 1-8 |
| Azt | Aztreonam | 4-16 | Lvx | Levofloxacin | 2-4 |
| Cfz | Cefazolin | 2-4 | Mer | Meropenem | 1-8 |
| Cpe | Cefepime | 8-16 | Mxf | Moxifloxacin | 0.5-1 |
| Cft | Cefotaxime | 1-2, 16 | Fd | Nitrofurantoin | 32-64 |
| Cft/CA | Cefotaxime/K Clavulanate | 0.5/4, 4/4 | Nxn | Norfloxacin | 4-8 |
| Cfx | Cefoxitin | 8-16 | P/T | Pipracillin/ Tazobactum | 16/4, 64/4 |
| Caz | Ceftazidime | 1, 4-16 | Tgc | Tigecycline | 1-2 |
| Caz/CA | Ceftazidime/ K Clavulanate | 0.25/4, 2/4 | To | Tobramycin | 4-8 |
| Crm | Cefuroxime | 4-16 | T/S | Trimethoprime/ Sulfamethexazole | 2/38 |
| Cp | Ciprofloxacin | 1-2 | - | - | - |
